# Low expression of neural cell adhesion molecule, CD56, is associated with low efficacy of bortezomib plus dexamethasone therapy in multiple myeloma

**DOI:** 10.1371/journal.pone.0196780

**Published:** 2018-05-08

**Authors:** Takashi Yoshida, Masaki Ri, Shiori Kinoshita, Tomoko Narita, Haruhito Totani, Reham Ashour, Asahi Ito, Shigeru Kusumoto, Takashi Ishida, Hirokazu Komatsu, Shinsuke Iida

**Affiliations:** Department of Hematology and Oncology, Nagoya City University Graduate School of Medical Sciences, Nagoya, Japan; Mayo Clinic Rochester, UNITED STATES

## Abstract

Bortezomib (Btz) is an active agent used to treat multiple myeloma (MM). Not all patients who receive Btz-containing therapy show a favorable response. Interaction of cellular adhesion molecules with MM and bone marrow stromal cells is crucial for the survival of MM cells. However, little is known about the role of these molecules in the sensitivity of MM to Btz-containing therapy. Thus, we evaluated the correlation between the level of cellular adhesion molecules in MM cells and the efficacy of Btz plus dexamethasone (Bd) therapy. The expression of the neural cell adhesion molecule gene (*NCAM*, also known as *CD56)*, *ITGA4*, *CXCR4*, and other genes were analyzed in 74 samples of primary MM cells collected from patients before they received Bd therapy. Of the eight genes tested, expression of *NCAM* was lower among patients who responded poorly to Bd therapy. *In vitro* expression of NCAM induced by transfection of MM cells enhanced their sensitivity to Btz treatment by causing accumulation of polyubiquitinated proteins. Our results indicate that expression of NCAM is associated with better response to Btz treatment and is a promising candidate biomarker for predicting response to therapies involving Btz.

## Introduction

Treatment of multiple myeloma (MM) has changed markedly with clinical use of proteasome inhibitors (PIs) and immunomodulatory drugs. Bortezomib (Btz), a PI that targets the beta 5 subunit of the 20S proteasome in MM cells, has significant anti-MM activity when combined with other agents, such as dexamethasone, alkylating agents, and immunomodulatory drugs. Adding dexamethasone to Btz therapy has been reported to be associated with improved responses to treatment by patients with progressive disease or disease that is refractory upon initial Btz monotherapy, including 13 of 74 evaluable patients (18%) in the SUMMIT study and 9 of 27 (33%) patients in the CREST study [1]. Based on the results of other studies, alkylating agents, such as melphalan and cyclophosphamide, are favored for combination with Btz therapy [2–4]. Additionally, combination of Btz with lenalidomide and dexamethasone (Ld) is reported to have significantly prolonged the OS of patients with MM who were ineligible for transplants from 64 months (with Ld therapy) to 75 months [5].

Btz is the first PI that has been accepted as a key drug for the treatment of MM, including newly diagnosed and relapsed and refractory cases. However, MM remains incurable, as MM cells gain resistance to anti-cancer drugs, including Btz, over the course of treatment. Moreover, not all patients respond favorably to Btz treatment; a fraction of patients exhibits a suboptimal or no response to Btz.

Several studies have associated the reported mechanism of Btz resistance with mutations in the proteasome, changes in degradation of endoplasmic reticulum (ER)-associated proteins, and overexpression of several factors, including insulin-like growth factor (IGF)–1, mucin 1 (MUC1), CD44, hepatocyte growth factor (HGF), MET, and amylase [6–8]. These results were obtained from *in vitro* analyses of artificially established, Btz-resistant MM cell lines. Therefore, the clinical utility of these results remains unclear.

Several studies have tried to predict the efficacy of therapies that combine Btz and dexamethasone (Bd) using clinical data from MM patients receiving such combined therapies. These studies focused on markers associated with the ER stress response factors, such as activating transcription factor (ATF) and X-box binding protein 1(XBP1) [9–11]. In those studies, low expression of XBP or ATF3 and ATF4 associated with poor response and short PFS with Bd therapy. However, these markers have not been validated in other studies and, thus, the reproducibility of these studies is uncertain.

Cell adhesion molecules that mediate the adhesion of MM cells to stroma cells play a critical role in cell adhesion-mediated drug resistance (CAM-DR) [12]. Thus, they may contribute to the mechanism of resistance to Btz therapy. Expression of VLA-4, a member of the integrin superfamily of adhesion receptors that is recognized as a main factor involved in CAM-DR [13], is regulated by Btz treatment in MM cells. VLA-4, also known as a CD49d, is highly expressed in MM cells, where it plays a critical role in resistance to conventional cytotoxic agents, such as vincristine, etoposide, and melphalan. Btz treatment suppresses expression of VLA-4 in MM cells, and therefore might rescue the sensitivity of MM cells to cytotoxic agents. Whether other adhesion molecules associated with sensitivity to cytotoxic agents are involved in the acquisition of resistance to Btz treatment by MM cells is unknown.

Neural cell adhesion molecule (NCAM), also known as CD56, belongs to the immunoglobulin superfamily. CD56 is characteristically expressed in MM cells, but not in normal plasma cells [14, 15]. Although this molecule is often used to diagnose aberrant plasma cells and residual disease after treatment [16], its role in the biology and survival of MM cells has not been fully investigated.

In the current study, we analyzed the basal expression of adhesion molecule-related genes in primary MM cells derived from patients receiving Bd therapy. Decreased expression of *NCAM* was seen among poor responders to Bd therapy. Furthermore, overexpression of *NCAM* promoted Btz-induced apoptosis in MM cells by enhancing ER stress caused by proteasome inhibition.

## Materials and methods

### Isolation of primary MM specimens and subsequent experimental conditions

All samples were obtained from Nagoya City University Hospital between January of 2007 and December of 2013. The Institutional Ethical Committee in Nagoya City University Hospital approved the study (approval number: 113). Seventy-four bone marrow (BM) specimens were collected from patients with MM prior to Bd treatment. Written informed consent was obtained from all patients. Primary MM cells were isolated from the bone marrow mononuclear cell fraction with anti-CD138 antibody-conjugated magnetic beads using an AutoMACS pro separator (Miltenyi Biotec, Auburn, CA), an automatic magnetic cell sorting system [17]. To minimize the effect of contamination with normal plasma cells, only those BM specimens for which clonal proliferation of MM cells was confirmed by both pathological diagnosis and flow cytometric analyses were used in this study. In addition, to standardize the experimental conditions, all BM specimens were immediately purified and stored.

All primary MM samples were subjected to mRNA extraction followed by quantitative polymerase chain reaction (PCR) to analyze the expression of the three translocation-related genes, *CCND1*, *FGFR3*, and c-*MAF*, as described previously [18]. Abnormal expression of any of the three genes was further evaluated of karyotype translocation involving t(11;14), t(4;14) or t(14;16) by fluorescent *in situ* hybridization.

MM cells were screened for expression of NCAM on their surfaces by flow cytometry analyses (SRL, Tokyo, Japan). MM cells were defined as CD38-positive cells expressing monoclonal cytoplasmic light chain. The ratio of NCAM-positive cells in the whole population of MM cells was calculated. Response to Bd therapy was assessed according to the International Myeloma Working Group Uniform Response Criteria 2006.

### Cell culture and reagents

Two human MM cell lines, KMS-11 and NOP-1, were cultured as described previously [19]. Btz was purchased from Wako Pure Chemical Industries (Osaka, Japan). Antibodies against ubiquitin, CHOP, and actin were purchased from Santa Cruz Biotechnology (Santa Cruz, CA). Antibodies against NCAM and cleaved caspase-3 were purchased from Cell Signaling Technology (Danvers, MA). Antibody against caspase-8 was purchased from BD bioscience (Franklin Lakes, NJ).

### Apoptosis assay

Apoptosis of cells exposed to Btz for 16 h was evaluated using annexin V-FITC and propidium iodide (Medical & biological laboratories, Nagoya, Japan). The fraction of positive cells was determined using a FACS Calibur (BD Bioscience, San Jose, CA). The percentage of specific apoptosis was calculated as follows: = 100 * (% induced apoptotic cells—% spontaneous apoptotic cells) / (100 -% spontaneously apoptotic cells). Cell proliferation assays were performed as described previously [20,21]. The IC_50_ value was calculated using GraphPad Prism version 6.05 for Windows (GraphPad Software, La Jolla, CA).

### Immunoblot analyses

MM cell lines were incubated with or without Btz for 6–12 h. Whole-cell extracts were prepared and analyzed as described previously [22]. Each loaded sample was adjusted to 30 μg of denatured protein per 10 μL using a Bradford protein assay. The results of the immunoblot analysis were representative data from duplicate experiments.

### Quantitative real-time reverse transcription polymerase chain reaction (PCR) analyses

Total RNA was extracted from purified MM cells stored at -80°C using the RNeasy mini kit (Qiagen, Valencia, CA). Reverse transcription and amplification of total RNA were performed using the CellAmp whole transcriptome amplification kit (Takara Bio, Shiga, Japan). This kit generates cDNA from mRNA using oligo dT-primers and facilitates uniform cDNA amplification by PCR. Quantitative PCR was carried out using SYBR green gene expression assay (Toyobo, Osaka, JAPAN) and a Step One Plus Real-Time PCR instrument (Applied Biosystems, Foster City, CA) according to the manufacturer’s instructions. Targeted primer sets used for quantitative PCR (*ITGA4*, *ITGAL*, *ICAM1*, *NCAM*, *CXCR4*, *CD44*, *HGF*, *MET* and *ACTB*) were purchased from Takara Bio. All samples were run in duplicate and gene expression was normalized to that of *ACTB*.

### Stable overexpression of NCAM by lentivirus transfection

A gateway entry vector encoding the open reading frame of *NCAM* was purchased from DNAFORM (DNAFORM, Yokohama, Japan) sublicensed from RIKEN (RIKEN, Wako, Japan). The lentivirus-based expression system was constructed as a combination of the entry vector, cytomegalovirus (CMV) promoter-containing vector, and plenti6.4/R4R2/V5-DEST multisite gateway vector using BP and LR reactions (Invitrogen, Carlsbad, CA). Lentiviruses were produced and harvested as previously described [22]. KMS-11 and NOP-1 cell lines were infected with NCAM-containing or LacZ-containing lentivirus for 24 h with polybrene, followed by incubation with 10 μg/μL blasticidin for 3 weeks. Overexpression of NCAM or LacZ in stable clones was confirmed by flow cytometry analyses. Antibody against NCAM conjugated to PE was purchased from BD bioscience.

### Stable knockdown of NCAM expression with lentiviral miRNA

miRNA targeting NCAM (ID: Hmi411197) was chemically synthesized, annealed, terminally phosphorylated, and inserted into an entry vector using BLOCK-iT™ Pol II miR RNAi Expression Vector Kits (Invitrogen, Carlsbad, CA). Ineffective sequences, designated “negative,” were used as controls. The lentivirus-based expression vector was constructed as a combination of the miRNA-containing entry vector, CMV promoter-containing vector, and plenti6.4/R4R2/V5-DEST multisite gateway vector using BP and LR reactions. In the same method used to establish NCAM-overexpressing clones, OPM-2 cells were infected with miRNA-containing lentivirus, and then NCAM knock-down cells were established.

### Statistical analyses

Statistical analyses were performed using GraphPad Prism software. Patient characteristics was compared with Mann-Whitney U test and chi-squared test, Gene expression was compared using the Mann–Whitney U test. Student t-tests were used to compare percentages of specific apoptosis; *p* < 0.05 indicated statistical significance.

## Results

### Characterization of MM patients receiving BD therapy

Between January 2007 and December 2013, all MM patients receiving Bd therapy in our institute were enrolled in the present study. They received an intravenous or subcutaneous dose of 1.3 mg/m^2^ Btz weekly or twice weekly. Of all 74 patients, 69 patients (93.2%) received Btz twice weekly, and 72 patients (97.2%) received 1.3 mg/m^2^ as their starting dose of Bd treatment. On the day of Btz treatment and on the following day, 20 mg of dexamethasone was administered orally or intravenously. Seventy-four patients from whom BM specimens were collected prior to the initial Bd therapy were chosen for analyses. Based on their responses to Bd therapy, these patients were classified as either good responders (CR + VGPR + PR, n = 56) or poor responders (SD + PD, n = 18). Patient characteristics are shown in Table 1. There were no significant differences in age and type of M protein between the two groups. The ratio of females was higher among the poor responders. Regarding prior treatment, poor responders were treated more heavily than were good responders (median = 2 vs. 1). However, the ratio of heavily treated patients, defined as 3 and over, in both groups was nearly equal. Analyses of cytogenetic/genetic risk category revealed that poor responders included a slightly larger population of patients with high-risk features (n = 7/18, 39.0%) than did good responders (n = 17/56, 30%). The median progression-free survival of good responders (171 days) was longer than that of poor responders (87.5 days).

**Table 1:** Patient characteristics.

Characteristic	Good responder	Poor responder	
Total number	56	18	
Median age (range)	67 (35–75)	63 (36–80)	0.7099
Sex			
male	32 (57%)	6 (33%)	0.1057
female	24 (43%)	12 (67%)	
M protein			
IgG	31 (55%)	6 (33%)	
IgA	11 (20%)	4 (22%)	
BJP	13 (23%)	5 (28%)	
IgD	1 (2%)	3 (17%)	
Light chain			
κ	37 (66%)	11 (61%)	0.7793
λ	19 (34%)	7 (39%)	
β2 microglobulin			
≥ 5.5 mg/L	23 (41%)	9 (50%)	0.5887
Albumin			
> 3.5 g/dL	31 (55%)	5 (28%)	0.0582
Cytogenetic/genetic aberrations			
t(11;14) / CCND1	8 (14%)	6 (33%)	
t(4;14) / FGFR3	12 (21%)	5 (28%)	
t(14;16) / cMAF	5 (9%)	2 (11%)	
Negative for above 3 translocations	21 (38%)	3 (17%)	
Not evaluated	10 (18%)	2 (11%)	
High-risk feature^†^	17 (30%)	7 (39%)	0.5676
Prior treatment			
0	14 (25%)	5 (28%)	
1	23 (41%)	1 (6%)	
2	9 (16%)	8 (44%)	
3 and over	10 (18%)	4 (22%)	0.7335
prior IMIDs‡	11 (20%)	7 (39%)	0.1199
prior PBSCT*	18 (32%)	5 (28%)	1.0000
Induction therapy for PBSCT*			
	10 (18%)	3 (17%)	1.0000
Best response			
CR	4 (7%)	-	
VGPR	24 (43%)	-	
PR	28 (50%)	-	
SD	-	15 (83%)	
PD	-	3 (17%)	
Progression-free survival (median)	171 days	87.5 days	

^†^High-risk feature includes the cases with t(4;14) /FGFR3 or t(14;16) /cMAF

‡ Including lenalidomide and thalidomide

*Peripheral blood stem cell transplantation

### Relationship between expression of cell adhesion molecule-related genes and response to Bd therapy

**Fig 1:**
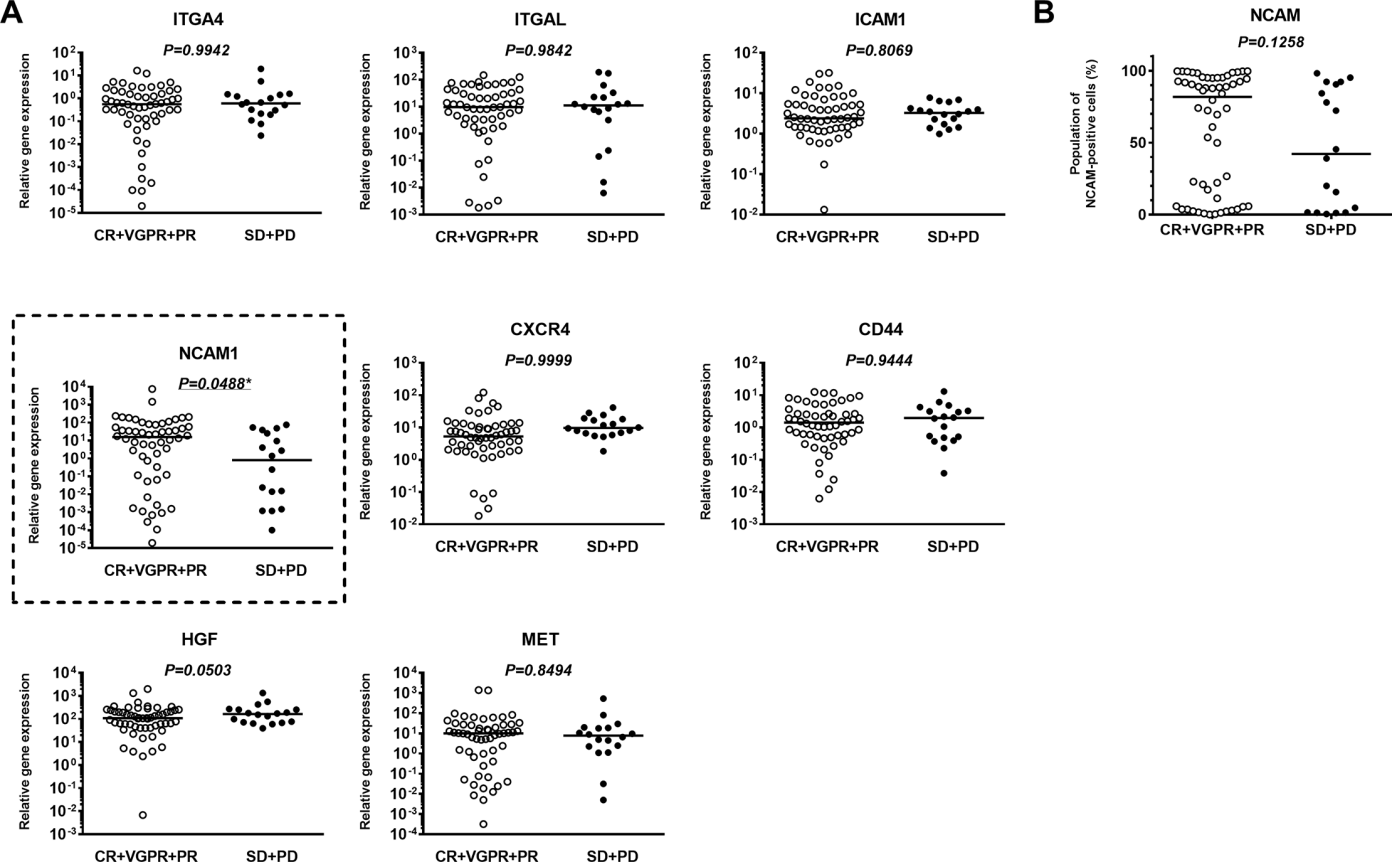
Comparison of levels of cell adhesion molecules between good and poor responders to bortezomib plus dexamethasone (Bd) therapy. (A) Comparison of the expression of several genes associated with cell adhesion molecules among good responders (CR+VGPR+PR) and poor responders (SD+PD) to Bd therapy. (B) Comparison of cell-surface expression of NCAM by FACS among the good and poor responders to Bd therapy. * indicates statistical significance (*p* < 0.05) as calculated by a Mann–Whitney U test.

Primary MM cells from 74 purified samples were subjected to quantitative PCR analyses (S1 Table). Expression of several cell adhesion molecule-related genes was measured and evaluated for association with response to Bd therapy. No significant differences were seen in the expression levels of seven genes: *ITGA4*, *ITGAL*, *ICAM1*, *CXCR4*, *CD44*, *HGF*, and *MET* (Fig 1A). The expression level of *NCAM* was significantly lower in poor responders than in good responders. Median values for *NCAM* mRNA in the poor and good responders were 0.82 vs. 15.62 (p = 0.0488), respectively (Fig 1A). In a comparison of cell-surface expression of NCAM analyzed by flow cytometry, there was no significant difference between poor responders and good responders (42.3% vs. 81.8%, p = 0.1258, Fig 1B).

**Fig 2:**
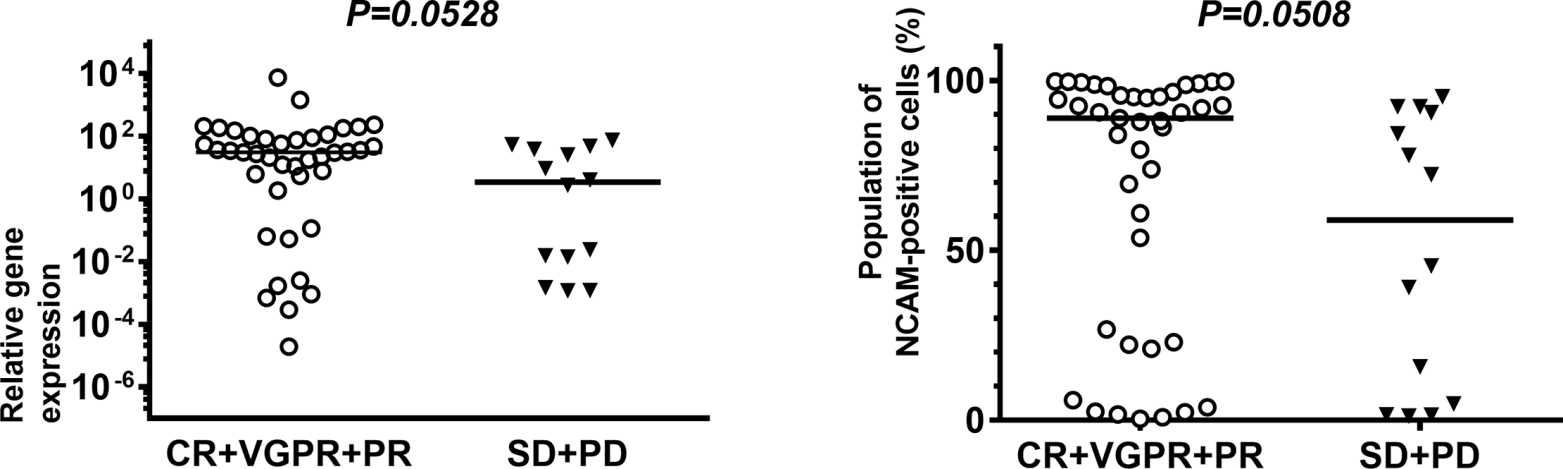
Comparison of levels of neural cell adhesion molecule (NCAM) among good and poor responders to bortezomib plus dexamethasone (Bd) therapy. Comparison of NCAM expression at the mRNA level (left side) and at the cell surface (right side) between the two groups, excluding seven t(14;16)-positive cases and 12 cases for which translocation of genes was not evaluated. * indicates statistical significance (*p* < 0.05), as calculated by a Mann–Whitney U test.

We have reported that t(14;16)-positive MM is negative for CD56 expression and leads to unfavorable outcomes, even after clinical use of PI and IMiDs [23]. All t(14;16)-positive MM samples were negative for NCAM expression in that study, wherein samples were defined as negative for expression of NCAM if less than 20% of the cells in the sample were NCAM-positive. Thus, to exclude the influence that NCAM-negative, t(14;16)-positive MM cases when assessing treatment response, we excluded 19 cases, including seven cases that were t(14;16)-positive and 12 cases that were not evaluated for the three gene translocations that typically occur with MM, and then re-evaluated the differences in NCAM expression among patients in the two groups. As shown in Fig 2, good responders tended to show an increased expression of *NCAM* at the mRNA level relative to poor responders (31.19 vs. 3.44, p = 0.0528). Good responders also tended to show a similar increase in the expression of NCAM at the cell surface relative to poor responders (88.90 vs. 58.85, p = 0.0508).

Using clinical data and data on gene expression in MM cells that were collected during the HOVON65/GMMG-HD4 trial (series GSE19784) and stored in the GEO database, a public functional genomics data repository, we evaluated the relationship between expression of NCAM and the efficacy of Btz-containing therapy [24]. Based on the data from that trial, patients who expressed lower levels of NCAM showed shorter PFS and OS than did patients who expressed higher levels of NCAM (S1 Fig).

### Expression of NCAM increases BTZ-induced apoptosis in MM cells

**Fig 3:**
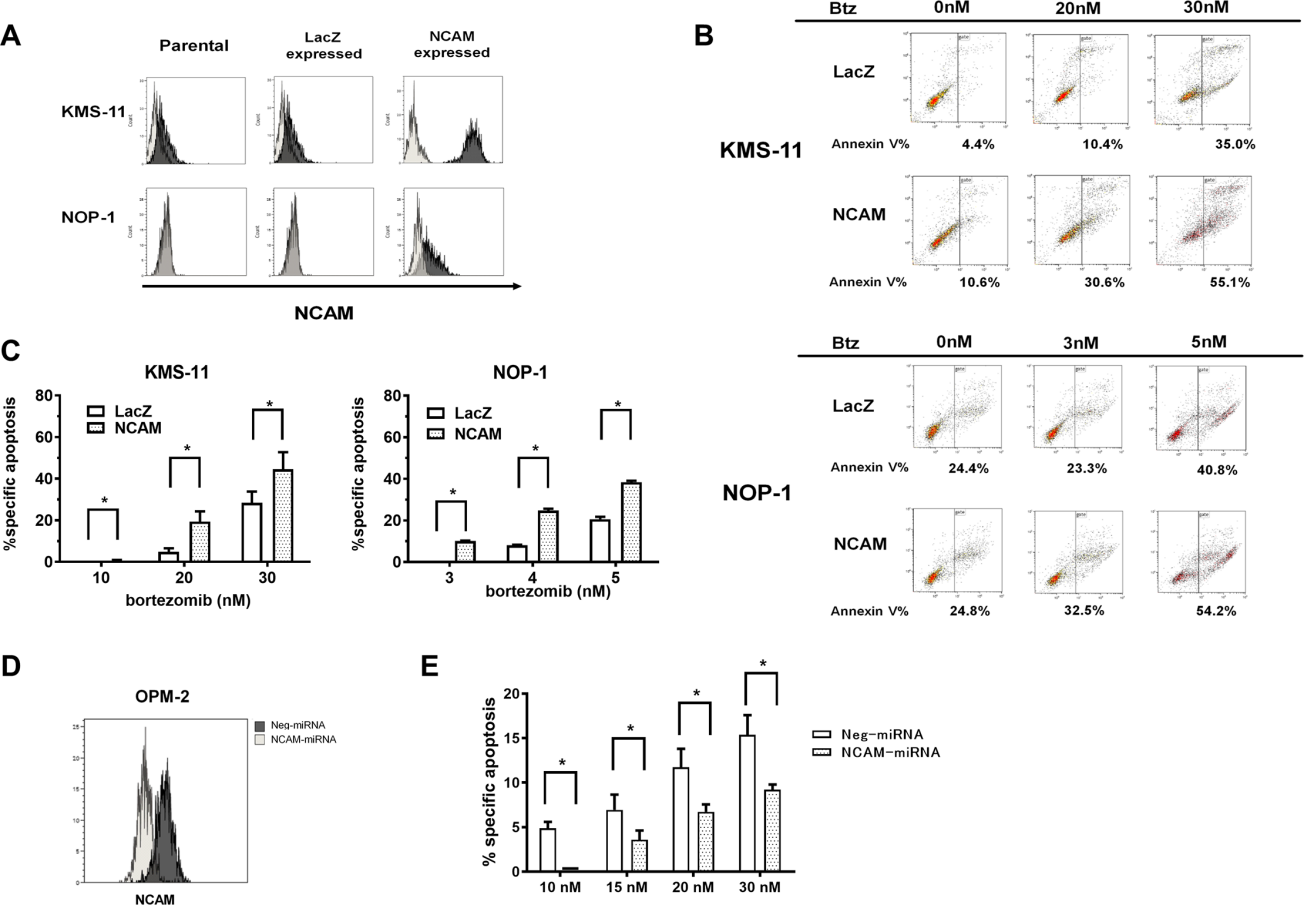
Comparison of rates of bortezomib (Btz)-induced apoptosis between cells with enhanced expression of neural cell adhesion molecule (NCAM) and LacZ-expressing cells. (A) Establishment of NCAM- or LacZ-expressing cells using two multiple myeloma (MM) cell lines, KMS-11 and NOP-1. Black area, CD56-PE; gray area, isotype. (B) Viability of NCAM- or LacZ-expressing cell lines after exposure to different concentrations of Btz for 16 h. (C) Evaluation of the percentage of NCAM- or LacZ-expressing cells undergoing specific apoptosis after exposure to Btz. (D) Establishment of NCAM knock-down cells using the OPM-2 cell lineage. Black area, cells transfected of negative (non-specific) miRNA cells; gray area, cells transfected to express NCAM-specific miRNA. (E) Evaluation of the proportion of NCAM knock-down cells undergoing apoptosis compared to that among control (miRNA-negative) cells after exposure to the indicated dose of Btz. * indicates statistical significance (*p* < 0.05), as calculated by student t-test.

To clarify the relationship between expression of NCAM and sensitivity to Btz treatment, two MM cell lines, KMS-11 and NOP-1, with low or no expression of NCAM were selected for further study. Using these cell lines, NCAM-expressing and LacZ-expressing cells (control) were established, and their levels of expression of NCAM and their promotion of Btz-induced apoptosis were analyzed. Both KMS-11 cells and NOP-1 cells transfected with lentiviral vector for expressing NCAM showed enhanced expression of NCAM relative to cells transfected with LacZ (Fig 3A). Btz treatment induced more apoptosis in KMS-11 cells expressing NCAM than in cells expressing LacZ, as indicated by the higher frequency of Annexin-V-positive cells among the former (Fig 3B).

Specific apoptosis in KMS-11 cells expressing LacZ or NCAM at a Btz concentration of 20 nM and 30 nM was 4.8% vs. 19.4% (p = 0.0084) and 28.5% vs. 44.6%, (p = 0.0465), respectively (Fig 3C, left). These differences in rates of specific apoptosis were statistically significant. Similar results were obtained in NOP-1 cell lines expressing LacZ or NCAM (Fig 3C, right).

As shown in Fig 3D, expression of NCAM was knocked down in OPM-2 cells using NCAM-specific miRNA. This downregulation of NCAM associated with reduced induction of apoptosis by Btz treatment (Fig 3E).

### Overexpression of NCAM enhances Btz-induced ER stress in MM cell lines

**Fig 4:**
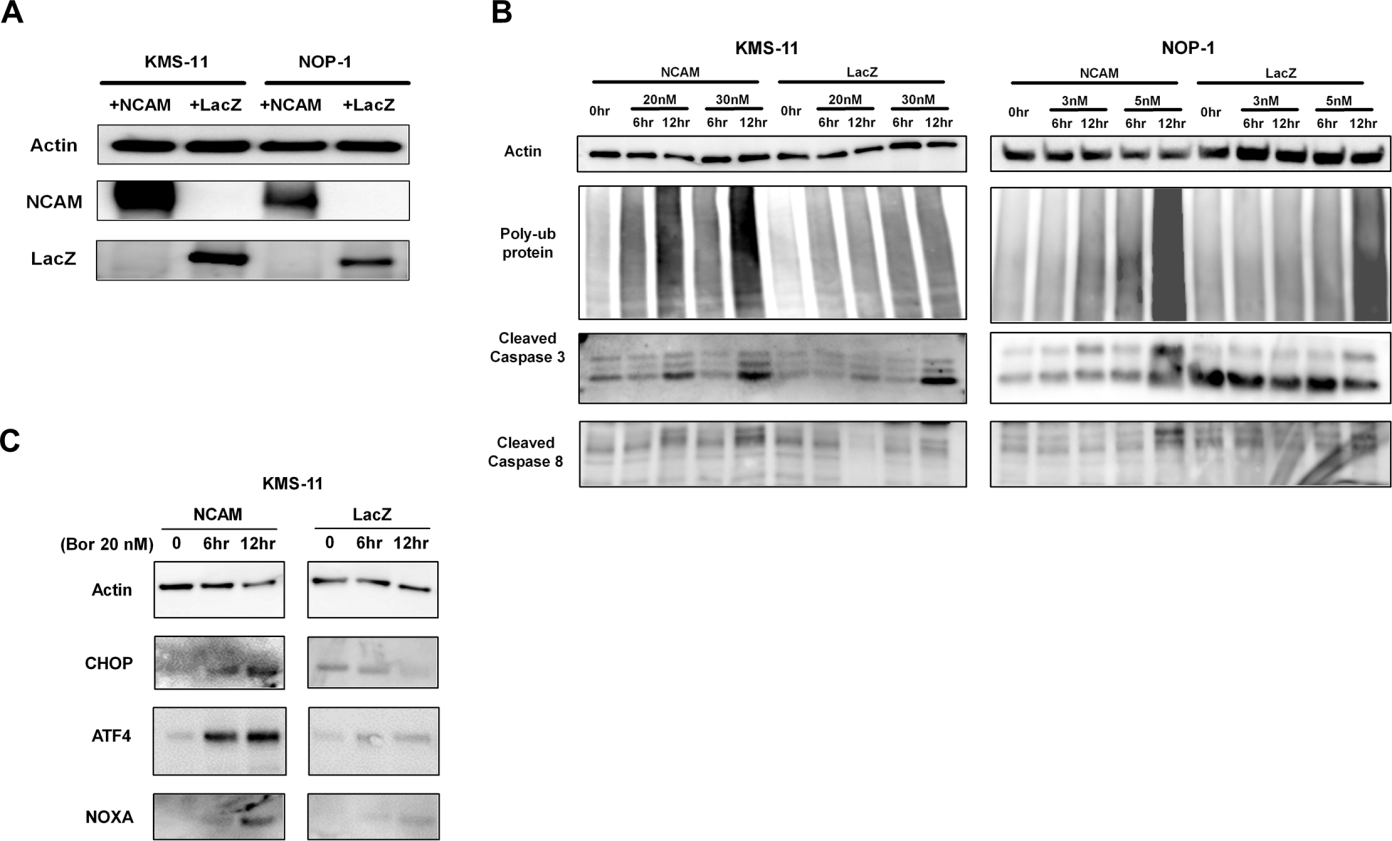
Altered expression of ER stress-related factors in cells expressing neural cell adhesion molecule (NCAM) or LacZ during bortezomib (Btz) treatment. (A) Expression of NCAM and LacZ in NCAM or LacZ expressing cell lines. (B) Altered expression of accumulated ubiquitin, caspase-3, and caspase-8 in NCAM- and LacZ-expressing cells during Btz treatment. (C) Altered expression of ER stress-associated factors, CHOP and ATF4, and NOXA in NCAM- and LacZ-expressing cells during Btz treatment.

Stable expression of NCAM and LacZ was confirmed in transfected KMS-11 and NOP-1 cells (Fig 4A). NCAM-expressing cells showed significant accumulation of polyubiquitinated proteins and enhanced expression of CHOP when treated with both 20 and 30 nM of Btz compared to LacZ-expressing cells, suggesting that expression of NCAM enhanced Btz-induced ER stress (Fig 4B). Twenty nM of Btz treatment induced caspase activation, as indicated by the enhanced expression of cleaved caspase-3 and caspase-8 in NCAM-expressing KMS-11 cells compared to LacZ-expressing control cells. Similar results were observed in NOP-1 cells treated with 3 and 5 nM Btz, respectively. These results suggest that expression of NCAM renders MM cells more sensitive to ER stress.

## Discussion

In the present study, we identified the influence of NCAM (CD56) on Bd therapy for MM. Enhanced expression of NCAM contributed to the improved anti-myeloma effect of Bd therapy by promoting ER stress upon Btz treatment.

NCAM is a neural adhesion molecule expressed in 70–80% of MM cases [15]. Lack of NCAM expression is reported to be a marker for poor prognosis when treatment options for MM are restricted to conventional chemotherapy [25]. However, the prognostic relevance of NCAM expression in regard to MM is also reported to decline for transplant-eligible patients when they are treated with high-dose chemotherapy followed by autologous stem cell transplant (ASCT) [26, 27]. In the era of novel agents, such as PI and IMiDs, the prognostic relevance of NCAM expression for patients with MM, including both those who are eligible for transplantation and those who are not, remains unclear.

Recently, Ying *et al*. retrospectively analyzed the association of expression of CD56 and CD117 by MM cells with prognosis in 50 patients with newly diagnosed MM that was treated with PI and/or IMiDs. Using multivariate analysis, they identified that high expression of NCAM is independently prognostic of longer overall survival [27, 28]. Our results indicate that NCAM is one of the factors involved in the sensitivity of MM to Btz-containing therapy. Therefore, whether MM cells in symptomatic patients receiving Btz-containing therapy express NCAM may have prognostic relevance. To confirm our results, further studies using larger populations are warranted.

Several studies have investigated the association of CD56 expression with the level of serum M protein in patients with MM, and based on their results, researchers have proposed that patients with CD56-positive MM cells tend to have higher levels of paraprotein than patients with CD56-negative MM [29,30]. These reports suggest that MM cells expressing CD56 are characterized by higher secretion of M protein followed by the overload of ER compared to CD56-negative MM cells. These MM cells are easily subjected to fatal ER stress under proteasome inhibition. This hypothesis is partly supported by our finding that NCAM-expressing MM cell lines accumulated more ubiquitinated proteins and exhibited excess ER stress upon Btz treatment compared to control cells. In our study, we tried to evaluate the association of paraprotein production levels in each MM case with CD56 expression in MM cells. However, no association was observed (data not shown). This may be due to variable cases of relapsed or refractory (RR) MM, the occurrence of which would reflect the heterogeneity of RR MM tumors.

We have proposed that t(14;16)-positive MM cases involving aberrant expression of MAF are characterized by poor prognosis, a high frequency of additional abnormal karyotypes, and frequent expression of CD20, along with several other karyotype and cytogenetic abnormalities. None of the cases involving a t(14;16) translocation were positive for CD56 expression, while all of those involving a t(4;14) translocation were positive for CD56 expression [23]. According to the results of our evaluation of MM cases that excluded t(14;16)-positive cases and cases not evaluated for genetic/cytogenetic abnormalities, good responders to Bd therapy expressed more NCAM than did poor responders, and the differences in NCAM expression between the two groups showed a trend towards a difference. Therefore, we speculate that NCAM expression could be one of the determining factors for sensitivity of MM to Btz treatment and propose that the correlation between expression of NCAM and cytogenetic abnormalities with regard to Btz sensitivity warrants investigation using a larger sample size.

In this study, enhanced expression of NCAM was found to associate with higher sensitivity to Btz treatment, as it triggered excess ER stress and enhanced Btz-induced apoptosis. In terms of cell-to-cell contact, NCAM is recognized as an adhesion molecule and is an important marker involved in the homing of MM cells [31, 32]. MM cell clones with lower expression of NCAM would have fewer interactions with other MM cells and/or stromal cells in the bone marrow, indicating weak addiction to the bone marrow milieu. The mechanism of action involved in the insensitivity of the clones with low expression of NCAM to Btz treatment is unclear. Weak addiction to bone marrow milieu has been linked with the epithelial mesenchymal transition (EMT) process or hypoxic condition, which accelerate the egress of MM cells to into the peripheral blood [33]. They seem to be associated with malignant transformation, such as in extramedullary disease and plasma cell leukemia, which are often observed during treatment of refractory MM. A high incidence of these malignant phenotypes is often reported in clinicopathological studies of NCAM-negative MM cells [25, 34, 35]. Further studies are needed to clarify the role of loss of NCAM expression in the emergence of malignant phenotypes during treatment.

In summary, we have shown that lower expression of NCAM associates with poor efficacy of Btz treatment. We have demonstrated that expression of NCAM enhanced the effect of proteasome inhibition and augmented Btz-induced apoptosis of MM cells. Although the level of NCAM in MM cells before treatment could potentially be used as a biomarker to predict the efficacy of Bd therapy or of other Btz-containing regimens, the clinical utility of evaluating expression of NCAM needs to be confirmed by larger-scale studies that include more samples of MM cells from patients showing resistance to Bd therapy. Also, to optimize a method for evaluating the NCAM expression, a comparison between gene expression and cell surface expression needs to be evaluated in large-scale studies. In addition, the level of NCAM expression in MM cells should also be further tested in patients receiving other agents, such as IMiDs, to determine whether the prognostic relevance of NCAM expression is specific to sensitivity to Bd therapy.

Our findings contribute to a better understanding of the role of NCAM in ER stress-related apoptosis caused by Btz treatment and suggest that NCAM expression may be a potential new marker for predicting sensitivity to Btz-containing treatment. The current study may also provide a new insight to predict the efficacy of next-generation proteasome inhibitors, such as ixazomib and carfilzomib.

## Supporting information

S1 Table Clinical features and expression of NCAM in MM cells. Summary of clinical features and expression of NCAM in MM cells derived from 74 patients receiving Bd therapy.
(XLSX)

S1 Fig Association of the level of NCAM expression with the outcome of Btz-containing therapy based on analysis of data from the HOVON65/GMMG-HD4 trial (GSE19784). The cut-off value for specifying low or high expression of NCAM was determined from the median value of the 280 samples. * indicates statistical significance (*p* < 0.05), as calculated by the log-rank test.
(TIF)
