# Supplementary figures and images for: Low expression of neural cell adhesion molecule, CD56, is associated with low efficacy of bortezomib plus dexamethasone therapy in multiple myeloma
Source: PLoS One. 2018 May 8;13(5):e0196780. doi: 10.1371/journal.pone.0196780 (PMC5940221; doi:10.1371/journal.pone.0196780)

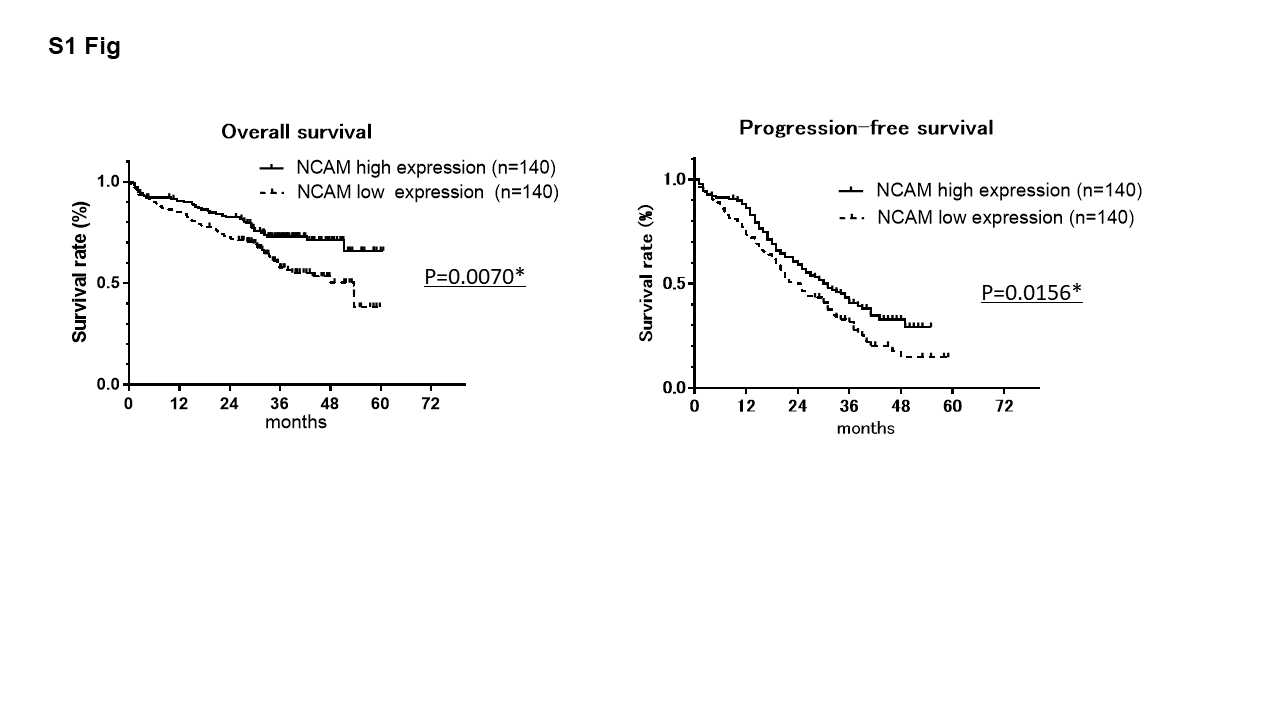

Supplement: S1 Fig — The cut-off value for specifying low or high expression of NCAM was determined from the median value of the 280 samples. * indicates statistical significance (p < 0.05), as calculated by the log-rank test. (TIF) [file pone.0196780.s002.TIF]
